# Abdominal Rigidity and Lower Extremity Weakness due to a Latrodectus Bite in Greece: A Case Report

**DOI:** 10.7759/cureus.83770

**Published:** 2025-05-09

**Authors:** Stefanos Votsis, Dimitrios Karapiperis, Georgios Sfikas, Christos A Papanastasiou, Anthimos Pehlivanidis

**Affiliations:** 1 Cardiology, Medical College of Georgia at Augusta University, Augusta, USA; 2 Cardiology, 424 Military Hospital, Thessaloniki, GRC; 3 Internal Medicine, 424 Military Hospital, Thessaloniki, GRC; 4 Cardiology, Aristotle University - School of Medicine, Thessaloniki, GRC

**Keywords:** arachneidae, black widow, latrodectism, latrodectus, latrodectus tredecimguttatus, poisoning, spider, spider bite

## Abstract

Latrodectus, or "black widow" spiders, are found worldwide. Black widow envenomations are rare occurrences in Greece. Black widow bites cause unremarkable local lesions that may also include systemic signs and symptoms. Our purpose is to present a rare case of a black widow spider bite.

Herein, we report the case of a 66-year-old farmer who presented to our hospital due to abdominal pain and was admitted after he was bitten by a black widow spider. Our patient showed the typical symptoms of latrodectism, that is, rubor and pain at the bite spot, washboard abdomen, rhabdomyolysis, and lower-extremities neuromuscular paralysis. Our case was complicated by acute renal failure, which is not common in such cases. A quite remarkable finding is the detection and capture of the culprit arthropod from the patient’s clothes and its identification by expert military veterinarian-arachnologists. The patient was completely cured and discharged after a week-long hospitalization.

Black widow spider bites, rare as they may be, especially in Greece, should be considered within the differential diagnosis of unremarkable local lesions, rhabdomyolysis, or acute renal failure.

## Introduction

Spider bites are rare medical events, and venomous spider bite cases are uncommon in Greece, despite the presence of *the Latrodectus* spider species ("black widow") in southern Europe [1]. Black widow spider bites cause unremarkable local lesions that are sometimes accompanied by systemic reactions. We report the case of a 66-year-old farmer who was admitted to our clinic after he was bitten by a black widow spider.

## Case presentation

A 66-year-old male farmer was transferred from a regional hospital to the Military Hospital of Thessaloniki because of intense abdominal pain and extreme perspiration. The symptoms' onset was acute and occurred while working in his field. Upon his arrival at the hospital, the patient had difficulty walking due to lower limb weakness and a “washboard” abdomen. He also mentioned symmetrical upper-limb numbness. Though the clinical picture of his hospital admission was "acute abdomen", the patient reported progressive alleviation of the pain, which initiated as back pain and gradually spread throughout the abdomen.

The patient’s medical history included hypertension and dyslipidemia. He also had a pacemaker, which was implanted two months earlier due to complete atrioventricular block. At his arrival, the patient had a blood pressure of 130/80 mmHg, O2 saturation of 94%, and his ECG demonstrated normal sinus rhythm with 100 beats per minute.

The laboratory admission test showed leukocytosis and mild renal insufficiency, which did not exist before admission according to the patient (Table 1). The patient's urine volume during the first 24 hours of hospitalization was marginal (800 ml) with an administered hydration volume of 2000 ml.

**Table 1:** Laboratory findings

Parameter	Reference Range	Hospital Day 1	Hospital Day 2	Hospital Day 3	Hospital Day 5
WBC (k/μL)	4.0-11.0	17.84	17.96	13.56	11.9
Ht (%)	42.0-54.0	44.2	43.2	43.9	48.9
PLTs (k/μL)	142-450	196	182	167	185
Urea (mg/dl)	17.97-55.0	50.6	58	72.5	116
Creatinine (mg/dl)	0.72-1.25	1.78	1.71	1.57	1.47
CPK (U/L)	30-200	105	201	-	460

**Figure 1:**
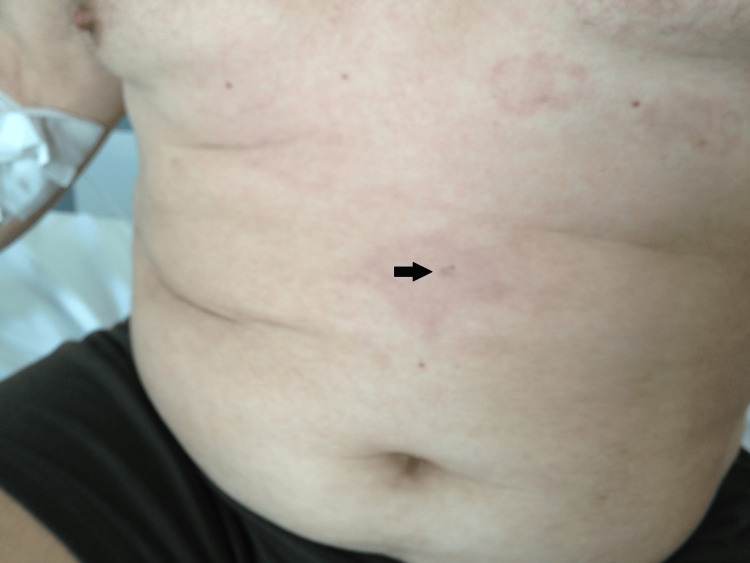
The patient's initial rash presentation. The presumed point of entry (“Fang sign”) can be detected at the center of the rash (black arrow).

During the second day of hospitalization, the patient displayed macular confluent rash on both sides of his lateral abdomen, which was migratory almost from the beginning, and a visible skin lesion 2 cm over his navel, which resembled an insect bite and was surrounded by a red halo (Figure 1).

By the second day of hospitalization, there was a gradual improvement in the neurological signs of the lower limbs, accompanied by the ongoing resolution of his neuromuscular weakness. Notably, he reported dysesthesia, manifesting as causalgia and mild pruritus, in the absence of any cutaneous lesions on the lower extremities. These sensory disturbances were not present initially.

**Figure 2:**
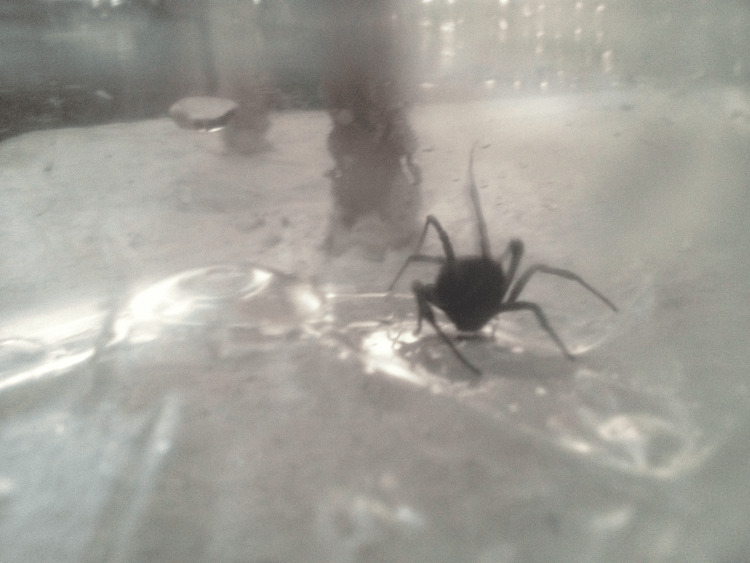
The spider captured from inside the patient's clothes.

**Figure 3:**
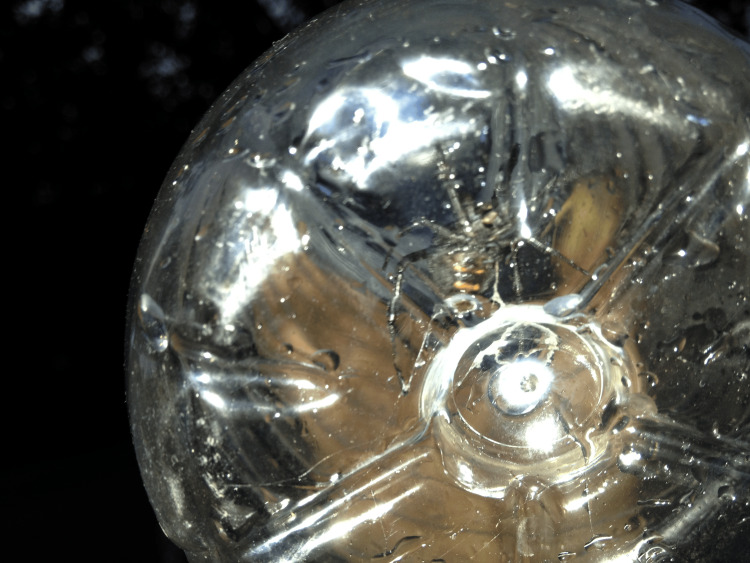
Another photo of the captured culprit spider. The characteristic orange stripes on the lower part of its body are clearly visible.

The patient's renal function slightly improved (creatinine 1.57 mg/dl), and accordingly, there was improvement of his urine output, which reached normal levels (1500ml/24h). WBC also improved (13,560/μlt). The low-grade fever continued. However, the key clinical element of the second day of hospitalization was the verification of the clinical suspicion of “insect-bite-like syndrome", as the patient’s relatives found and promptly captured a small black spider from the clothes worn by the patient at the time of the pain onset. The culprit, which was about 2 cm in size, was collected alive (Figures 2-3) and sent for identification at the Military Veterinary Hospital of Thessaloniki.

On the third day of hospitalization, the clinical condition improved significantly: the “washboard” abdomen resolved; at the same time, the lower limb weakness almost vanished, and the patient regained his mobility. The lateral abdominal rash relocated a few centimetres from its original position and at the same time extended to the inner surface of the right arm. The supra-umbilical skin lesion, which we considered to be the bite site, had lost its discoloration. The patient's creatinine improved further (1.47 mg/dl), while the leukocytes reduced to normal levels. However, despite the patient’s normal hydration levels, we observed creatine phosphokinase (CPK) elevation without clinical symptoms (myalgia). CPK started to decline the very next day of hospitalization and was restored to normal levels within the next three days.

A week after his admission to the hospital, the patient reported no symptoms, the erythema had fully resolved, and renal function was restored. The treatment of our patient included adequate hydration, analgesia, and administration of antihistamines.

Our patient did not receive the customary antivenin (Aracmyn Plus) for two reasons: first, the clinical picture began to improve the day after hospital admission, and second, the antivenin used in such cases has not yet been approved by the EOF (National Organization for Medicines). Moreover, it was not deemed necessary to hospitalize the patient in the ICU for 24-hour monitoring because the improvement of his clinical state was already visible from the second day of hospitalization.

In our case, the spider was captured alive by the patient’s son, who found it inside the clothes worn by the patient at the moment of the bite. The spider was approximately 2 cm long, had a glossy black color, and two orange "stripes" on its abdominal region. Its size and color were suggestive of a female "black widow" spider, and its original, informal "identification" from internet images was identical to the final specialist parasitological report.

The official result of the spider examination by the Special Laboratory of the Military Veterinary Hospital of Thessaloniki is as follows: an Arachnid arthropod of the family Araneidae, genus* Latrodectus*, species *Latrodectus tredecimguttatus* (common name: black widow).

The spider bite incident described is one of the few observed in recent years in Greece and probably the only one in which there is full confirmation of the diagnosis due to the discovery and capture of the arthropod inside the patient’s clothes.

## Discussion

Cases of venomous spiders’ bites are not common in Greece, although the spider species black widow inhabits Southern Europe [1]. The classic black widow is usually of bright black color, with a characteristic red mark on its abdomen and one to one and a half centimetres in size. Its bite is not always perceptible and is rarely lethal. Systemic envenomation can become gradually apparent, causing pain that extends beyond the bite site.

The cause of the clinical picture is a specific neurotoxin (a-latrotoxin) which acts by paralyzing presynaptic neurons, resulting in increased release of neurotransmitter substances [2].

*Latrodectus* envenomation may include: acute pain at the bite site with characteristic erythema ("Fang sign"), washboard abdomen, excessive sweating, myalgia, rhabdomyolysis, and neuromuscular paralysis of the lower limbs [3,4]. When the bite site is located at the abdomen, the pain is so acute that it is often confused with an acute abdomen. The characteristic erythema at the bite site becomes visible between the first and second day after the bite and usually lasts four to six days. Necrosis rarely develops at the bite site. Seldom, the clinical picture can be complicated, as in our case, by acute renal failure.

The disease treatment involves initially symptomatic treatment (cleaning the area with soap and water, ice application and elevated limb positioning to reduce the absorption of venom, and potential hospitalization depending on severity and comorbid conditions) and then, rarely, potential administration of the specific antivenin [5], which, however, is implicated in (sometimes severe) allergic reactions. For the treatment of patients with painful muscle spasms caused by the spider toxin, anticonvulsants, benzodiazepines, and opiates are administered. Rarely, antibiotics are also used when contamination of the bite site is probable. In case necrosis develops, debridement is recommended. The disease’s mortality is fortunately very low. Only three fatalities have been reported so far internationally [6].

## Conclusions

Black widow spider bites should be considered within the differential diagnosis of unremarkable local lesions, rhabdomyolysis, or acute renal failure. In our case, the final diagnosis of latrodectism was confirmed by the capture and identification of the culprit arthropod, which was found inside the patient’s clothes, very shortly after his transfer to our hospital.
